# How Are People Undergoing Dialysis Expected to Benefit From Cognitive Behavioural Therapy? A Realist Analysis

**DOI:** 10.1111/hex.70466

**Published:** 2025-10-11

**Authors:** Katrin Micklitz, Joanne Greenhalgh, Lori Suet Hang Lo, Richard Sawatzky, Kara Schick‐Makaroff

**Affiliations:** ^1^ Faculty of Nursing, College of Health Sciences, Dianne and Irving Kipnes Health Research Academy University of Alberta Edmonton Alberta Canada; ^2^ School of Sociology and Social Policy University of Leeds Leeds UK; ^3^ School of Nursing Trinity Western University Langley British Columbia Canada; ^4^ Centre for Advancing Health Outcomes Providence Health Care Research Institute Vancouver British Colombia Canada; ^5^ Institute of Health and Care Sciences, and Centre for Person‐Centered Care (GPCC), Sahlgrenska Academy University of Gothenburg Gothenburg Sweden

**Keywords:** cognitive behavioural therapy, depression, dialysis, mood disorders, psychological stress, psychosocial intervention

## Abstract

**Introduction:**

Depressive symptoms remain inadequately addressed and undertreated in people who receive life‐prolonging dialysis treatment. Cognitive behavioural therapy (CBT) has been shown to be effective for treating depression; however, we lack an understanding of how and under what circumstances people with depressive symptoms receiving dialysis may benefit from it. The aim of this study is to identify ideas underlying CBT in general and develop an initial programme theory that explains how these ideas might apply to people receiving dialysis. It is the first step of a theory‐driven explanatory realist synthesis and realist evaluation.

**Methods:**

This study included a broad literature search and interviews with seven CBT therapists across Canada and the United States. Search terms were derived from CBT and refined to theory‐based literature, literature reviews and book chapters. Therapists were recruited through team collaborators and had experience in developing or providing CBT to adults with depressive symptoms, including those receiving dialysis. Qualitative analysis of data from the literature and interviews focused on identifying mechanisms through which CBT is expected to reduce depressive symptoms in people receiving dialysis and the circumstances that may shape these mechanisms.

**Results:**

Based on our findings from 30 documents and the interview data, individuals living with dialysis treatment and experiencing depressive symptoms may benefit from CBT through (1) cognitive changes related to their illness and self; (2) experiencing pleasant emotions; and (3) feeling seen, understood and accepted. However, people's capacity to engage with CBT may be limited due to significant illness and treatment burdens, as well as the perceived stigma of mental health issues. Our findings can be explained by the cognitive behavioural model, illness adjustment theories such as the common‐sense model of self‐regulation, response shift theory, client‐centred therapy, and the cumulative complexity model.

**Conclusion:**

This study contributes to knowledge by explaining how the illness context of dialysis treatment might shape the mechanisms through which CBT is expected to work. Understanding the dialysis illness context when developing psychosocial interventions such as CBT can advance the provision of person‐centred mental health kidney care.

**Patient or Public Contribution:**

This patient‐oriented research leveraged established partnerships including a Community Advisory Committee, an equity, diversity, inclusivity (EDI) champion, industry partner, kidney administrators and clinicians, and CBT experts. The Community Advisory includes 10 people who have met monthly for over 10 years; the Committee itself is co‐chaired by a person with lived experience. The Community Advisors collaborated on the original study idea, participated in grant proposal development, gave feedback on ethics applications and study design, provided input on the initial programme theory, and co‐presented at provincial Nephrology Grand Rounds and Research days. They continue to lead in the next phases of this project.

## 1 Background

**Figure 1:**
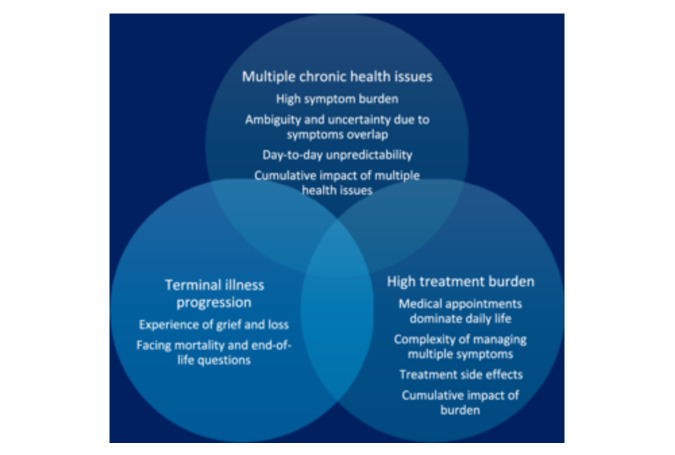
Contexts of people living with dialysis treatment.

Despite the high prevalence of depressive symptoms [1] and some of the poorest quality of life among those living with chronic illnesses [2], mental health concerns remain inadequately addressed and undertreated in people undergoing life‐prolonging dialysis treatment [3, 4, 5]. Cognitive behavioural therapy (CBT) is a frontline non‐pharmacological treatment for depression and has been recommended for depression in adults with chronic physical health problems [6, 7]. Studies have shown that CBT can reduce depressive symptoms in people undergoing dialysis [8, 9, 10, 11, 12, 13]; however, we lack insights into how and under what circumstances they may benefit from it. Living with dialysis is a complex condition, typically involving multiple chronic symptoms, significant treatment burdens, and a progressive, terminal course, each bringing its own potential challenges and experiences (Figure 1). Moreover, depressive symptoms in people receiving dialysis often overlap with uraemic symptoms, making it difficult to distinguish between psychological distress and the medical condition itself [14]. Likewise, CBT is a complex intervention that encompasses multiple therapeutic strategies, extends over a length of time, and requires active engagement from participants. To consider CBT as an effective psychosocial intervention for people undergoing dialysis, a better understanding is needed of how its therapeutic strategies interact with the specific dialysis illness context, as well as how improvements in mental health manifest in that population. The need for our research project was first raised by people receiving dialysis and kidney practitioners. In a kidney patient‐led study on mental wellness [15], some of our Community Advisors noted that ‘the most glaring gap in care is the lack of psychosocial support available to patients’ (p. 3).

We chose a realist evaluation approach because it addresses complexity and potentially provides explanatory insights into how and in what circumstances an intervention ‘works’ [16]. The present study is the first step of a larger research project, seeking to explain how, why, for whom, and under what circumstances CBT reduces depressive symptoms in individuals undergoing dialysis [17]. Following realist research methodology [18, 19], the aim of the present study is to identify key ideas underlying CBT in general and develop an initial programme theory (IPT), which is a middle‐range theory that explains how these ideas might apply to people undergoing dialysis. This IPT will then be tested and refined in the subsequent steps of our realist synthesis and realist evaluation.

### 1.1 A Realist Understanding of Complex Interventions

To deliver solutions for real‐world practice, the Medical Research Council's framework for developing and evaluating complex interventions [20] recommends that researchers should not only assess whether the intended outcomes of complex interventions are achieved, but also how and under what circumstances these outcomes are realised [20]. This involves developing theories about how an intervention may interact with various contextual factors to generate different causal mechanisms and outcome patterns. Realist research sets out to do just that. It is a theory‐driven, explanatory approach that seeks to identify the mechanisms through which an intervention achieves its effects and the contexts in which these mechanisms become active [21]. The output is a realist programme theory that explains how and under what circumstances an intervention works, expressed as context–mechanism–outcome configurations (CMOCs). A realist programme theory is a middle‐range theory, further tested and refined in future studies [22].

**Figure 2:**
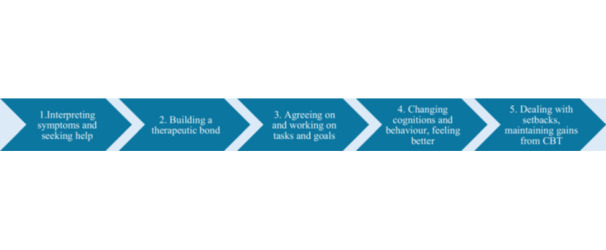
Process of change in CBT.

Realist research is based on the following principles: First, it is not the intervention itself that generates outcomes but the ways individuals choose to engage with it. An intervention provides resources, and individuals respond to these resources cognitively, emotionally and behaviourally, based on their individual dispositions, as well as situational, interpersonal, institutional and societal factors. In realist research, these responses are considered mechanisms [23]; they are the underlying and often invisible forces that generate outcomes. Second, interventions are conceptualised as processes involving several stages of change, each requiring individuals to make decisions [19]. In CBT, these decisions include interpreting symptoms and seeking help, building a therapeutic bond, agreeing and working on tasks and goals, changing cognitions and behaviour, dealing with setbacks, and maintaining gains (Figure 2). Each decision involves mechanisms that likely contribute to the observed outcomes [19]. Third, mechanisms do not work universally but are shaped by context, including a person's characteristics and capacities; intervention properties; interpersonal relations; and broader social, economic, institutional and cultural norms and settings [24]. In other words, context is inextricably linked to the mechanisms through which an intervention works [25]. CBT is an example of an intervention that has been applied in many different illness contexts. To develop the IPT, we explored how the specific contextual features experienced by those undergoing dialysis might shape how they engage with the intervention and influence the mechanisms through which it is expected to work (Table 1).

**Table 1:** Glossary of realist terms.

Term	Definition
Context	Context encompasses the conditions and circumstances under which mechanisms become active. This includes a person's characteristics and capacities, programme properties, interpersonal relations as well as the broader social, economic, institutional and cultural norms and settings. In realist evaluation, contexts cannot be understood independently of a mechanism; it is the special condition that shapes and modifies a particular mechanism generating the outcome of interest.
Mechanism	In realist evaluation, mechanisms are the underlying forces that generate outcomes. Mechanisms are not necessarily identical to those proposed by the ‘official’ programme theory. A programme offers resources, and how these are used depends on an individual's choices and their capacities to act on these choices. Moreover, mechanisms are context‐sensitive, meaning they are activated only in specific contexts. In summary, mechanisms in this study are understood as an individual's cognitive, emotional or behavioural responses to the resources provided by CBT, leading to various outcomes.
Outcome	Outcomes result from the interaction between contexts and mechanisms. They can range from a person's involvement with CBT to various mental health and well‐being outcomes and behavioural changes, and they may include intended and non‐intended, proximal and distal outcomes. (Informed by [20, 23])

## 2 Methods

**Figure 3:**
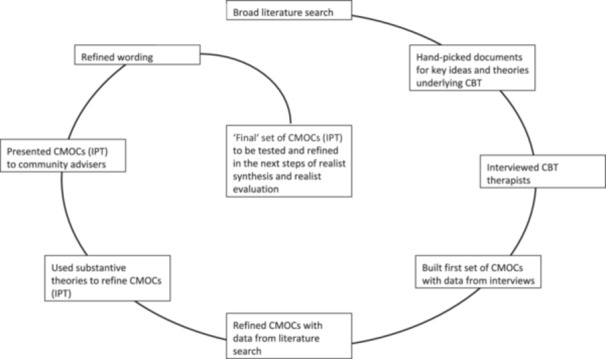
Process of initial programme theory (IPT) development.

The first step in realist research involves identifying key theoretical ideas underpinning an intervention and articulating initial theories that contain tentative ideas of how context might shape the mechanisms through which an intervention produces outcomes [18]. These theoretical ideas will be formulated into an IPT that will then be tested and refined in the subsequent steps of a realist synthesis and/or realist evaluation. To discern key ideas of CBT and develop initial theories about how these might apply to individuals receiving dialysis, we conducted (1) a broad literature search and (2) interviews with CBT therapists. (For an overview of the process of IPT development, see Figure 3.) Using complementary data sources, such as published literature and primary data from practitioners, is common practice in realist theory development [26].

### 2.1 Data Collection

#### 2.1.1 Literature Search and Screening

To identify theories and evidence that facilitated our understanding of how and why CBT works for different populations, with the support of a health sciences librarian, we conducted a broad literature search in OVID EMBASE and Scopus. The search terms, focused on theory‐related literature and results, were limited to books, book chapters, umbrella reviews, meta‐analyses and Cochrane reviews (Supporting file 1a). We also screened websites of psychological associations and institutions (Supporting file 1b). From our search, we purposively selected a sample of documents to gain an overview of the key ideas and assumptions underlying CBT before screening all remaining articles for additional theories to help us understand (1) how CBT works in general and (2) why and for whom it may ‘work’ in dialysis care. Through citation tracking, we added relevant documents for theory building. During screening, we included literature focused on individual CBT delivered in person or remotely to adults in general or adults with depression, fatigue, long‐term or incurable conditions, comorbidities, and dialysis‐related mental health issues. We excluded literature on specific mental health conditions, group or self‐help CBT, and mindfulness‐ or acceptance‐based CBT due to the potential for different underlying mechanisms. Further details on our literature search are provided elsewhere [17, 27].

#### 2.1.2 Interviews With CBT Therapists

We conducted semi‐structured, in‐depth interviews with CBT therapists, informed by initial findings from our preliminary analysis of the papers identified by our literature search. The purpose of these interviews was ‘theory gleaning’, which aims to obtain a set of ideas about how an intervention may work and what circumstances may shape its working. Practitioners generally have theoretical and tacit knowledge about the intervention and the circumstances that are more supportive of its success or failure [28]. To learn from a wide range of practitioners, we recruited therapists with different clinical backgrounds and expertise.

We developed an interview guide based on recommendations for conducting realist interviews at the stage of theory gleaning [28] (Supporting file 2). The interviews started with general questions about the therapists' background and approach to delivering CBT. Next, we asked what the therapists considered the most important outcomes in CBT for depression (exploring outcomes), what they believed led to these outcomes (exploring mechanisms), and for whom CBT was likely more effective (exploring contexts). When therapists had experience in working with people undergoing dialysis, we explored how outcomes, underlying CBT processes, and supportive circumstances differed from those in other populations. Finally, we presented ideas from the literature and previous interviews and invited the therapists to comment based on their knowledge and experience (probing theories). This realist interview technique is known as the ‘teacher‐learner cycle’, where interviewer and interviewee share and discuss their ideas about how and in what circumstances an intervention works [28]. We stopped recruiting additional therapists once no new ideas were presented in the interviews.

Interviews lasted between 45 and 90 min and took place from August to October 2023. They were conducted, recorded and automatically transcribed via Zoom, a videoconferencing application. Transcripts were manually cleaned of errors, false starts, repetitions, filler words and confidential information.

### 2.2 Data Analysis and Synthesis

The analysis and synthesis drew on data from the identified literature and the interviews. We applied a realist lens using the CMOC as an analytical tool. CMOCs are a heuristic in realist research used to formulate theories about how contexts are expected to modify mechanisms to generate outcomes [24, 29], and they are typically written as ‘*If … then…*’ hypotheses. We created a first set of CMOCs in a spreadsheet and mapped them along the stages of change (Figure 2). We then uploaded all transcripts and included literature into NVivo, a software for organising and analysing qualitative data, where we created a code for each stage of the change process, and within these stages, a code for each CMOC. We iteratively refined our initial CMOCs and built new ones by pooling data from the transcripts and the literature. We used ‘substantive theories’ (i.e., well‐established theories from particular domains such as psychology) to make sense of parts of our data, refine some CMOCs, and shift our theories to more abstract levels. The use of substantive theories is recommended for realist theory building, as it helps identify mechanisms and contexts and connects the findings to existing knowledge [24]. Coding and theory building were performed by one team member (K.M.), and the results were regularly shared and discussed within the team (K.S.M., J.G. and L.S.H.L.).

### 2.3 Patient and Public Involvement

We presented our IPT to six of our 10 Community Advisors during an in‐person patient engagement workshop in July 2024. Theories were presented in plain language and modified based on notes taken from the Advisors' feedback.

### 2.4 Ethical Approval

Ethical approval for this study was granted from the University of Alberta (ID Pro00129407) and Trinity Western University (ID 23EA03).

## 3 Results

**Figure 4:**
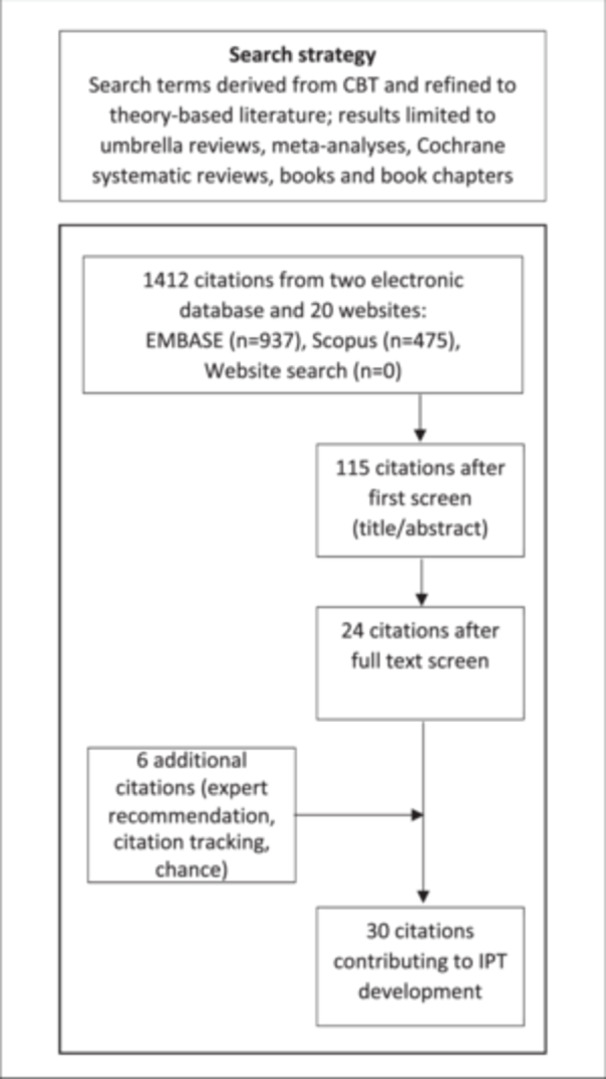
Flow diagram of broad literature search for initial programme theory (IPT) development.

Our results are based on 30 documents from the literature (Figure 4) and interviews with seven CBT therapists (Table 2). In what follows, we first summarise the CBT model of therapeutic change as described in the literature. We then present five theories—developed from the interviews with CBT therapists and supported by findings from the literature—regarding how the CBT model of therapeutic change may apply to the dialysis illness context and what other mechanisms might affect CBT outcomes for people undergoing dialysis (Table 3).

**Table 2:** Overview of all interviewees.

Interviewee (therapist)	Country	Gender	Race	Ethnicity	Background	CBT experience	Current work setting	Experience with individuals receiving dialysis
A	Canada	Woman	South Asian	Indian	Psychologist	8 years	Private practice Public	No
B	The United States	Man	White	Jewish	Psychologist	20 years	Outpatient	Yes
C	The United States	Woman	Latin American	Mexican	Social worker	3 years	Outpatient Public	Yes
D	Canada	Woman	White	Eastern European/Nordic	Registered therapist	25 years	Private practice	No
E	Canada	Man	White	English	Psychologist	—	Outpatient Public	Yes
F	Canada	Man	White	Eastern European	M.D., Mental health generalist	30 years	Public	No
G	The United States	Woman	—	White	—	—	—	Yes

**Table 3:** Overview of initial programme theory.

**Theory**	**Label**	**Initial programme theory**	**Substantive theories to support IPT**
Theory 1	Cognitive changes related to illness and self	In people with multiple chronic health issues (C1) and terminal illness progression (C2), modifying unhelpful cognitions related to illness and self may reduce depressive symptoms (O) as it increases hope (M1), control (M2) and illness coherence (M3). It may also allow individuals to adjust to lower levels of functioning (M4), modify their expectations of what is important in life given their new circumstances (M5), reframe certain aspects of their identity (M6), find meaning and benefit in their new situation (M7), discover purpose within the new circumstances (M8), and/or refocus on aspects in their life that remain within their control (M9). Different illness stages (C3) may require different cognitive strategies.	CBT model of therapeutic change (cognitive restructuring) Illness adjustment theories such as the common‐sense model of self‐regulation [30] and response shift theory [31, 32, 33]
Theory 2	Experiencing pleasant emotions	For people receiving dialysis whose lives often revolve around managing illness (C), engaging in activities that support pleasant emotions–such as joy (M1), meaning (M2), purpose (M3), reward (M4) and mastery (M5)–may enhance overall well‐being (O1) and encourage further health‐promoting behaviours (O2) through positive feedback loops (M6).	CBT model of therapeutic change (behaviour activation) Reinforcement theory
Theory 3	Feeling seen, understood and accepted	For people living with an illness characterised by high levels of ambiguity and uncertainty related to symptoms and illness progression (C1) as well as loss, and grief (C2), feeling seen, understood and accepted may improve well‐being (O). This is because it offers hope for improvement (M1) and supports individuals in processing their fears, worries (M2) and experiences of loss and grief (M3). Skilled and experienced therapists (C2) may be better equipped to provide such support because they have seen a wide range of emotions and are able to hold the space.	Client‐centred therapy (C. Rogers ‘empathy’ and ‘unconditional positive regard’) [34, 35]
Theory 4	Capacity to engage with CBT	For people receiving dialysis who face high illness and treatment burdens (C1), the demands of CBT might create a workload–capacity imbalance (M) hindering involvement with therapy (O1). While personal (C2) and socio‐demographic (C3) factors may further exacerbate the perceived imbalance between workload and capacity, strong self‐efficacy beliefs (C4) as well as a therapist's responsiveness and adaptability to the person's condition and needs (C5) may help reduce it and hence promote involvement with CBT (O2).	Cumulative complexity model [36]
Theory 5	Normalising conversations about mental health	In a dialysis illness context where the focus is on managing and treating disease (C1), avoiding the label ‘depression’ and normalising conversations about mental health might make it more acceptable (M) for people to seek psychosocial support when needed (O). Stigma of mental health issues may be more prevalent in certain cultural communities (C2) and among older generations (C3), as well as in healthcare settings that are based on a biomedical model (C4).	Common‐sense model of self‐regulation [30]

### 3.1 CBT Model of Therapeutic Change

The CBT model of therapeutic change (hereafter ‘CBT model’) is based on the notion that emotional distress is a consequence of maladaptive cognitions (i.e., unhelpful automatic thoughts, core beliefs and schemas) and that altering these cognitions leads to symptomatic change (cognitive mediation theory) [37, 38, 39, 40]. In people with depression, maladaptive thoughts commonly revolve around a person's self‐image, their perception of the world, and their expectations for the future [41].

Key elements in changing maladaptive thoughts in CBT are cognitive and behavioural interventions, such as cognitive restructuring and behaviour activation. In addition, CBT typically includes psychoeducation, that is, a structured way of providing information to help an individual understand distress and what generates and sustains it. A therapist might also teach practical problem‐solving skills and breathing or relaxation techniques to strengthen a person's coping. CBT is based on a collaborative approach where the therapist and the client work as partners to identify issues, set goals, examine and test thoughts and beliefs, and develop strategies to manage or change unhelpful thoughts and behaviours.

Although CBT is grounded in well‐formulated theories, what exactly leads to symptomatic change is not fully understood; cognitive mediation theory is contested [40, 42, 43, 44]. More specifically, it remains unclear whether cognitive change is necessary for (lasting) positive CBT outcomes and how this occurs. An alternative CBT theory suggests that depressive symptoms improve as a result of experiencing joy, reward, mastery or meaning [37, 38, 44, 45] brought about by behavioural interventions and potentially creating positive feedback loops and gain spirals (reinforcement theory) [37].

### 3.2 Towards CBT in a Dialysis Illness Context

The CBT model outlined above is expected to work universally, with only minor variations depending on the specific mental health condition. Much emphasis has been placed on delivering CBT as intended, in a standardised way, through the provision of training and manuals [46]. However, this ignores the context in which CBT is delivered, how it is delivered, by whom and to whom. There has been considerable debate and research about which characteristics of therapists and clients and the interactions between them and environmental factors are associated with CBT outcomes (Supporting file 3). For example, individuals have been shown to benefit more from CBT when they have sufficient cognitive capacities [47, 48] and learned resourcefulness [42], actively engage in the homework [42, 44, 49], and when therapists maintain a symptom‐focused approach [42, 49] and adhere to the treatment manual [44]. In contrast, individuals with cognitive dysfunction [44, 48] and severe psychological problems [44], as well as those living in socio‐economically deprived circumstances with stressors that may not be resolved through therapy [50], have been found to benefit less from CBT. Most importantly though, contextual factors are often positioned as ‘noise’ either interfering with the delivery of therapy as intended or leading to unwanted variation in therapy outcomes. However, from a realist perspective, these contexts are important explanatory features that can illuminate how the context of therapeutic delivery can shape the mechanisms through which the therapy works [25]. The literature is missing an elaboration of how these contextual features modify the mechanisms through which CBT works and the outcomes it produces.

Notably, some literature suggests that individualising the treatment to the specific client and taking account of their illness context is associated with improvements in therapy [49], especially in people with more complex and long‐term conditions [38, 51]. This challenges the idea that CBT should be delivered in a standardised way and raises questions about what features of the illness context are important in shaping the mechanisms of CBT in a dialysis context. Our interviews with CBT therapists sought to address these questions and move towards more explanatory theories about how and in what circumstances individuals undergoing dialysis may benefit from CBT.

The first three theories focus on *how* individuals receiving dialysis may benefit from CBT and the other two on the *circumstances* that may shape involvement with therapy. There are overlaps between theories. All theories combine data from the interviews and included literature and are illustrated by selected interview quotes. (Supporting file 4 provides an overview of all 21 CMOCs including sample data to support our theories.) To prevent readers from connecting individual comments to a specific interviewee‐therapist, quotes are not linked to pseudonyms.

Theory 1 Cognitive changes related to illness and self (CMOCs 1–4)

In people experiencing depressive symptoms, CBT typically targets maladaptive cognitions related to the self, the world and the future [41]. For individuals receiving dialysis, it may be helpful to target these maladaptive cognitions through the lens of people's illness experience. Living with dialysis can substantially affect an individual's self‐concept. For instance, being forced to stop working due to illness may threaten the identity of someone regarded as the family provider. Or, when illness dominates a person's life, their sense of self may become so closely intertwined with the illness that they come to see themselves solely through the lens of their ‘sick’ label. Such associations between the illness and the self can contribute to the development of depressive symptoms.

To regain a positive outlook on themselves and the world and adjust to the changes brought on by illness, individuals can use various cognitive strategies; CBT can help them to identify, develop and apply these strategies [52, 53]. One strategy is changing one's illness perceptions. According to Leventhal's common‐sense model of self‐regulation (CSM) [30], individuals cope with illness in ways that are shaped by their illness perceptions—that is, by their views about the illness's identity, causes, consequences, controllability and expected timeline. CBT can support individuals in developing more adaptive illness perceptions, thereby promoting more effective coping and enhancing overall well‐being. For example, if they feel overwhelmed by their multiple health issues, they may identify symptoms they can influence through their behaviour:

There was a lot of other health issues going on, and a lot of things that he couldn't control. His vision was failing, he had arthritis, he was having difficulty with daily living tasks … a lot of things he couldn't control because of his health. And so, we talked a lot about trying to focus on the things that he could and changing that.

Interviewee‐therapist

This way, they might recover a sense of hope and control, both of which have been associated with positive outcomes in CBT for depression [40, 42, 54, 55, 56]. A therapist may further support individuals in making sense of their symptoms, thereby reducing fear [45] and pathologisation [38, 55, 57, 58] and enhancing overall illness coherence. Modifying illness perceptions based on Leventhal's CSM was one strategy incorporated into the development of an online self‐help CBT programme in the included literature aimed at improving distress in people undergoing dialysis [38].

Another cognitive illness adjustment strategy is to adjust one's internal standards, values or conceptualisations in response to illness. Such an adjustment could lead to a phenomenon known as ‘response shift’, that is, a change in the meaning of one's self‐evaluation of a target construct [31, 32, 33]. For instance, individuals may adjust to lower levels of functioning (recalibration), modify their expectations of what is important in life given their new circumstances (reprioritisation), or reframe the target construct (reconceptualisation). An athletic person whose mobility is suddenly severely limited by illness may, for example, redefine what it means to be physically active:

They tend to only see themselves as dialysis patients … they tend to forget that there's other things in their life that they're able to do. So, it's … having that change of mindset that just because I can't run anymore doesn't mean that I can't do anything.

Interviewee‐therapist

Similarly, a person could redefine what constitutes depressive symptoms (e.g., a person may come to see that somatic symptoms such as ‘feeling tired’ or ‘having poor appetite’ can be depressive symptoms). Lastly, people may learn to find meaning and benefit in their new situation, discover purpose within the given circumstances, and refocus on aspects of their life that remain within their control [52]. Different illness contexts might require different strategies. It has been argued that people with severe conditions and poor prognosis may benefit less from changing illness perceptions, as their situation may offer fewer aspects that can be reframed [52]. Cognitive reframing may also be less effective when the causes of depressive symptoms are biomedical rather than psychosocial [59], as one interviewee noted:

Sometimes … they're just pretty sick … so their mood is about as optimized as it can be, and the reason why they have high scores or they're reporting not feeling good is really because of the medical side of what's going on.

Interviewee‐therapist

In summary, CBT might reduce depressive symptoms in people receiving dialysis through modifying maladaptive cognitions related to their illness and themselves. This theory is supported by illness adjustment theories, such as the CSM, and response shift theory.

Theory 2 Experiencing pleasant emotions (CMOCs 5–7)

Another CBT theory suggests that depressive symptoms improve as people experience pleasant emotions through behaviour activation, which encourages engaging in enjoyable and meaningful activities. In support of that theory, all interviewees emphasised that behaviour activation plays an important role in CBT for people receiving dialysis. As these people's lives are often dominated by dialysis and low mood, engaging in activities can reintroduce moments of joy, vitality, purpose and meaning [38]:

I had this one guy I worked with … big, burly football player, military fellow … and he was in a wheelchair, he was an amputee … he would go out to the porch and watch the hummingbirds at the hummingbird feeder, and … he was transfixed … and he would talk about it and filling the feeder and the water and the sugar … it was beautiful, and that was a way for him to be engaged in something that was meaningful and valuable to him.

Interviewee‐therapist

*Other pleasant emotions include reward and mastery, for instance, when people do things that they previously believed to be impossible, such as going for a brief walk or changing their diet. Pleasant experiences might act as catalysts for sustained health‐promoting behaviours (reinforcement theory), potentially creating positive feedback loops and gain spirals*.

Therapists might also support individuals in finding practical ways to continue living a meaningful life and experiencing joy [38]. They may teach techniques like stretching, progressive muscle relaxation, deep breathing, mindfulness, visualisation and self‐affirmation, all of which can promote relaxation and other positive states, thereby potentially helping people cope with pain, anxiety and stress (though they may also provide distraction and a sense of control) [45, 54]. In summary, individuals living with dialysis may benefit from CBT through engaging in activities that allow them to experience pleasant emotions like joy, meaning, purpose, reward and mastery.

Theory 3 Feeling seen, understood and accepted (CMOCS 8–12)

Apart from mechanisms linked to cognitive and behavioural CBT activities, mechanisms such as feeling seen, understood and accepted may play an important role in improving the emotional well‐being of people receiving dialysis. These mechanisms are linked to the therapist's empathy and unconditional positive regard, both of which are considered forceful elements in psychotherapy [44, 54, 56, 60], particularly in Rogers' client‐centred therapy [34, 35]. In CBT, empathy and unconditional positive regard are seen to enhance the likelihood of therapeutic change by strengthening the therapeutic alliance and paving the ground for in‐depth cognitive interventions [54, 56, 60], a notion that was univocally shared by our interviewees. Beyond that, they may contribute in a distinct way to positive outcomes in CBT for individuals receiving dialysis.

First, if people feel seen and understood, they may gain reassurance that their symptoms, thoughts and emotions are not out of the ordinary. This can occur when therapists show that they comprehend the complexities involved in living with dialysis treatment. An important feature of the dialysis illness context is that individuals often experience high levels of ambiguity and uncertainty related to their symptoms and illness progression. They feel helpless and hopeless. An empathetic therapist might give them the confidence that their experiences are not unusual and that something can be done to make them feel better. Therefore, the interviewees argued, it was crucial for therapists to show empathy and tailor sessions to the unique needs and concerns of the dialysis population. In the literature, a tailored CBT approach has been found to be particularly effective for individuals with long‐term conditions and medically unexplained symptoms [51]. Interviewees also noted that empathy and unconditional positive regard required therapists to be experienced, as their exposure to a wide range of emotions enables them to recognise and remain present with difficult emotions more easily. In the literature, therapist competence has been found to be linked to better CBT outcomes [42, 44] and is assumed to be particularly important for treating complex psychological conditions [41].

Second, people often do not know with whom to talk about the emotional impact of living with dialysis treatment. Many do not want to burden their families and friends, and they often do not receive much emotional support from medical professionals who may not see it within their scope nor know how to give this kind of support. CBT can provide a safe environment for individuals to express their fears and worries [38], thereby providing relief and facilitating emotional processing. Notably, and in alignment with CBT pioneer A. Beck [58], two interviewees argued that, strictly speaking, emotional release was not a component of CBT. Nevertheless, they acknowledged that sometimes it was the only activity people receiving dialysis had the capacity to engage in during CBT sessions.

Third, feeling seen, understood and accepted might play an important role in working with loss, grief and death, which are important features of living with dialysis. One interviewee talked about the importance of recognising the emotional work involved in coming to terms with grief and death, allowing people to reconnect with their humanness and thereby improving their quality of life:

The grief work, the end‐of‐life work, the wrestling with hard topics, the levels of anxiety were very high. An integrated behavioral healthcare of some sort within that setting, I think, would have improved quality of life…. I think they would feel seen, I think they would feel heard, I think they would feel like a human first and a patient second.

Interviewee‐therapist

The same interviewee acknowledged though that few CBT therapists were trained in grief and end‐of‐life support, so these topics may not be addressed in CBT for depression. In our interviews, only two interviewees brought up death, and both said it was not part of their CBT work.

In summary, for people living with an illness characterised by heavy emotional burden, loss and grief, feeling seen, understood and accepted may promote hope that they can do something to improve their situation, as well as help them process their fears and worries in the face of loss and grief.

Theory 4 Capacity to engage with CBT (CMOCS 13–17)

This and the next theory consider the implications of a dialysis illness context for an individual's involvement with CBT. CBT is a high‐intensity intervention [7]. It requires participants to be active agents in the change process and therefore have a certain level of self‐efficacy [57, 61]. In addition, participants must be able to access thoughts; describe emotional experiences; self‐explore; maintain focus [61]; have sufficient resources to attend sessions; and engage with in‐between tasks. All of these capacities may be compromised in people who undergo dialysis due to the nature of the illness and treatment context.

First, some interviewees observed diminished self‐efficacy beliefs in people with long‐term conditions, noting that those who have received much of their treatment in biomedical settings may have come to view medication and surgery as the only remedies, and those with a history of unsuccessful treatments may have lost trust in the possibility of improvement. However, the interviewees also insisted that CBT could restore people's self‐efficacy beliefs.

Second, due to medical issues, medication and dialysis itself, individuals may struggle to concentrate and engage with cognitive CBT activities, requiring therapists to include repetitions and visualisations, advance in small steps, and prioritise behavioural over cognitive techniques [45]. Third, in people with multimorbidity, other more pressing health issues might take centre stage during therapy sessions, calling for immediate attention [45]:

Sometimes they were dealing with so many health issues, so dialysis was just a very small part; they had to manage doctors' appointments for diabetes and had to see physical therapy and they were hospitalized…. They couldn't really focus on anything else other than just putting out little fires.

Interviewee‐therapist

In such situations, therapists will need to be able to respond flexibly to the person's circumstances and needs. Lastly, individuals who already juggle multiple medical appointments will likely find it difficult to make time for CBT sessions. Delivering CBT chairside during dialysis was seen by one interviewee as a way to accommodate this difficulty.

In some cases, the burden of living with dialysis adds to other existing difficulties, such as financial struggles, often linked to losing the ability to work, or the lack of social support—accumulating to a point where the demands of CBT become impossible. Studies have shown that people with socio‐economic difficulties tend to benefit less from CBT [50]. However, some individuals may overcome external challenges. For example, one interviewee recalled a person receiving dialysis who had been homeless previously and had no social support, but their strong self‐efficacy beliefs compensated for the lack of these resources, and they still benefited from CBT.

The complex interplay between high illness/treatment burdens, psychological and socio‐economic difficulties, and available resources to engage in CBT could be explained by the cumulative complexity model [36], another substantive theory that helped us identify and theorise potential mechanisms in CBT for people receiving dialysis. The model posits that self‐care and health‐related outcomes result from the balance (or imbalance) between the workload of demands (including medical appointments, self‐care, job and family) and the capacity to meet these demands (including self‐efficacy, physical and mental functioning, financial means, and social support). In other words, outcome patterns in CBT for people receiving dialysis may be explained by the *experience* of a workload–capacity balance or imbalance, which is, in turn, affected by the intersection of personal, socio‐demographic and illness‐related factors.

Theory 5 Normalising conversations about mental health (CMOCs 18–21)

Stigma surrounding mental health was brought up by all but one interviewee as a reason why people receiving dialysis may be reluctant to seek psychotherapy. Stigma can be actual or perceived, and interviewees found it to be more prevalent in certain cultural communities, such as Hispanic or Asian populations, particularly among older generations.

Interviewees found that stigma plays a complex role in integrated kidney care settings where healthcare professionals collaborate across disciplines to attend to a person's physical, psychological and social needs. Some individuals might avoid disclosing emotional struggles (e.g., by signing up for CBT), worrying they may be perceived as complicated or ungrateful, which could negatively affect their overall care [45]. In contrast, when stigma is less of an issue, people may engage in impression management differently—showing that they take proactive steps to manage their health [45] by signing up for CBT and letting others know. These patterns suggest that the dialysis environment and culture shape whether individuals open up about their distress and seek psychological help or not.

Consequently, interviewees emphasised the need to normalise conversations about mental health by incorporating them into routine dialysis care [45]. They further suggested that stigma may be overcome by referring individuals to psychotherapists through trusted medical experts [59] and avoiding labels such as ‘depression’ when offering CBT to people receiving dialysis:

People didn't like some of the pathologizing words that CBT sometimes used. People don't like ‘depression’, they don't like that word, they don't wanna think of it as a psychiatric illness or a mental health condition. They didn't like those terms; they wanted to use less stigmatizing language.

Interviewee‐therapist

Again, the CSM (see Theory 1) provides a useful framework for understanding individuals' decisions to sign up for CBT based on their views of the illness label ‘depression’. In summary, avoiding the label depression and normalising conversations about mental health in an illness context where the focus is on managing and treating disease might make it more acceptable for people to seek psychosocial support when needed.

#### 3.2.1 Patient and Public Involvement

Community Advisors agreed with the above five theories and helped us refine our wording to reflect the experiences of people receiving dialysis. In addition, they pointed us to two further explanations of why people receiving dialysis may not seek CBT: (1) They do not know what CBT is, nor if they might benefit; and (2) they do not want to be perceived as having yet another health problem on top of their existing multiple health issues.

Text box: Summary of initial programme theory (also see Table 3)

Individuals living with dialysis treatment and experiencing depressive symptoms may benefit from CBT through (1) cognitive changes related to their illness and self; (2) experiencing pleasant emotions; and (3) feeling seen, understood and accepted. All three mechanisms might strengthen hope, control and meaning in people receiving dialysis who typically experience a high degree of ambiguity and uncertainty due to multiple health issues, as well as grief and loss in the face of a terminal illness progression. They can also restore a sense of being fully human in people whose lives are often reduced to being a patient. These mechanisms may either directly reduce people's depressive symptoms or indirectly improve their emotional well‐being as they gain internal resources to engage in more health‐promoting behaviours.

However, in a dialysis illness context characterised by significant illness and treatment burdens, individuals may lack the capacity to engage fully with CBT, requiring therapists to adjust their therapy delivery and content flexibly and ongoingly. In addition, stigma around mental health issues can be a barrier to the uptake of CBT. Normalising conversations about mental health in dialysis care can make it more acceptable for people receiving dialysis to talk about their emotional issues and address their mental well‐being.

## 4 Discussion

The aim of this study was to identify key ideas underlying CBT in general and develop initial theories that explain how these ideas might apply to people receiving dialysis. We sought to examine how the specific contextual features experienced by people receiving dialysis might shape involvement with CBT and the mechanisms through which CBT reduces depressive symptoms. In the literature, the CBT model was presented as a universal model. It was largely assumed that CBT works the same for everyone and everywhere, depending on the specific mental health condition and provided that individuals have sufficient emotional and cognitive capacities to participate in cognitive restructuring. While the literature acknowledged that various contextual factors can impact the effects of CBT, we found no theories that explain how these factors influence the way people respond to CBT and shape outcome patterns. Only one paper from the included literature [38] offered theories about how people receiving dialysis might benefit from CBT, hypothesising that changing people's illness perceptions may reduce distress in that population.

Our interview data supported the notion that CBT outcomes in people receiving dialysis may be explained by changes in illness perceptions. Cognitive adjustment strategies could also lead to response shift due to recalibrating, reprioritising and reconceptualising, for example, how they view their depressive symptoms. Alternatively, people may feel better after CBT because they engaged in activities that allowed them to experience pleasant emotions and encouraged further health‐promoting behaviours through positive feedback loops (reinforcement theory). Lastly, people receiving dialysis may benefit from CBT because they feel seen, understood and accepted by a therapist who shows empathy and unconditional positive regard. Empathy and unconditional positive regard are not considered core components of CBT but rather general therapeutic qualities and cornerstones of client‐centred therapy [34, 35]. Therefore, their role in CBT for people receiving dialysis might be overlooked. However, our interview data suggest that feeling seen, understood and accepted can be particularly important for those dealing with the ambiguity, uncertainty, loss, and grief involved in living with dialysis.

**Figure 5:**
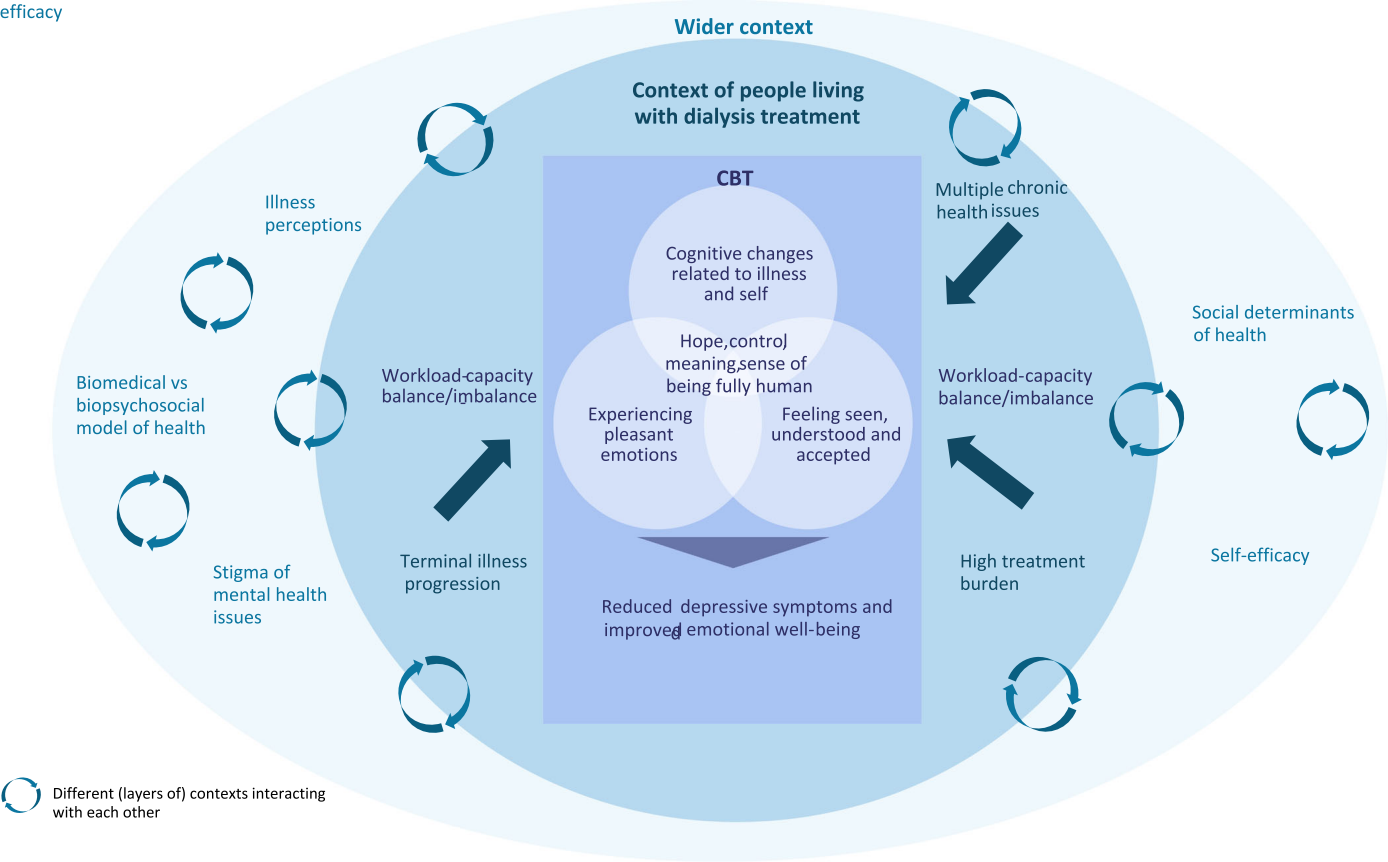
CBT for people receiving dialysis with depressive symptoms—Initial programme theory (IPT).

We further identified contextual features that may hinder the involvement with CBT in the dialysis illness context: the experience of an imbalance between high illness/treatment burdens and the capacity to meet the demands of CBT, as well as the stigma of mental health issues. We also outlined how these mechanisms may, in turn, be shaped by the wider context, such as personal, interpersonal, institutional and societal factors, and thereby addressed some of the complexities involved in providing CBT to people receiving dialysis. Our findings are expressed as an IPT (see Text box, Table 3 and Figure 5).

To identify and articulate potential mechanisms in CBT for people receiving dialysis, we used various substantive theories. The CSM [30] and response shift theory [31, 32, 33] helped us conceptualise potential cognitive changes after CBT as part of the illness adjustment process. The CSM may additionally provide a more comprehensive framework to explain whether or not people engage with CBT depending on how they perceive the illness label ‘depression’. The CSM is supported by a substantial body of research that has established a link between illness perceptions and coping behaviours/mental health outcomes in individuals with chronic [62, 63] or mental [64] illnesses. It is further backed by studies demonstrating a link between illness perceptions and depression in individuals during the first year of dialysis [65] and between illness perceptions and self‐care behaviours in people with chronic kidney disease [66]. We used Rogers' notion of client‐centred therapy [34, 35] to articulate our theory on feeling seen, understood and accepted. Finally, the cumulative‐complexity model [36] helped us interpret data related to high illness and treatment burdens and identify the experience of a workload–capacity balance/imbalance as a potential mechanism that explains levels of involvement with CBT. The cumulative‐complexity model is supported by research showing how cumulative medical, psychosocial and demographic difficulties negatively affect the uptake, involvement and effectiveness of mental health services [67]. It should be noted that the substantive theories we employed were not identified through a systematic literature search [68] but through some of the included documents and the knowledge of individual team members. There may be other substantive theories that explain better how and in what circumstances CBT benefits people receiving dialysis. Alternative theories may be identified in later stages of our realist synthesis and realist evaluation.

Our IPT remains tentative and will be subject to ongoing development. By choosing to focus on the dialysis illness contexts, we did not consider theories related to individual differences (e.g., personality structures and attachment issues) other than self‐efficacy. Furthermore, we did not have sufficient data to develop theories about how gender and ethnicity might modify the mechanisms through which CBT achieves its outcomes. Only one document from the included literature [44] noted that CBT outcomes may differ for people from different ethnic backgrounds and sexual orientations, however, without providing any further explanations. When we asked therapists in our interviews for whom they thought CBT was likely more effective, they were careful not to generalise or stereotype specific populations and emphasised that, with adaptations, CBT could work for everyone. As such, our analysis lacked theories about how social determinants of health (SDOH) might impact CBT outcomes beyond affecting a person's workload–capacity balance (e.g., when people experience existential difficulties) and apart from culturally rooted stigma of mental health issues. It must be noted, though, that this shortcoming is likely to be due to our search strategy not having included relevant terms related to SDOH. Our theories might differ depending on how long a person has been receiving dialysis and their depression trajectories [65]. Our data did not allow us to develop theories at this level of granularity. We also had no data on how individuals may handle setbacks and maintain CBT gains, and therefore, we lack theories for this stage of the intervention process. We hope to address these shortcomings in the next steps of our study as we test and refine our IPT. Our Community Advisors also shared tentative insights on why people receiving dialysis may not sign up for CBT, which we will explore in the next steps of our realist evaluation.

## 5 Conclusion

This study contributes to knowledge by explaining how the illness context of dialysis treatment may shape the mechanisms through which CBT is expected to work. Individuals undergoing dialysis often struggle with multiple chronic health issues, a heavy treatment burden and terminal illness progression. Taking these illness contexts and the wider social context (e.g., the medical system and culture) into account when developing psychosocial interventions such as CBT can advance us in providing person‐centred mental health kidney care.

## Registration Details

This study is part of a realist synthesis that has been registered with PROSPERO (CRD42023476184).

## Author Contributions

**Katrin Micklitz:** writing – original draft, investigation, methodology, visualisation, project administration, formal analysis. **Joanne Greenhalgh:** conceptualisation, writing – review and editing, methodology. **Lori Suet Hang Lo:** writing – review and editing, project administration. **Richard Sawatzky:** conceptualisation, writing – review and editing. **Kara Schick‐Makaroff:** conceptualisation, funding acquisition, writing – review and editing, project administration, supervision, methodology.

## Ethics Statement

Ethical approval for this study was granted from the University of Alberta (ID Pro00129407) and Trinity Western University (ID 23EA03).

## Conflicts of Interest

The authors declare no conflicts of interest.

## Supporting information

Clean Revised Supplementary Material.

## Data Availability

Qualitative interviews did not have ethical approval for the sharing of data.
