# Supplementary material for: How Are People Undergoing Dialysis Expected to Benefit From Cognitive Behavioural Therapy? A Realist Analysis
Source: Health Expect. 2025 Oct 11;28(5):e70466. doi: 10.1111/hex.70466 (PMC12514452; doi:10.1111/hex.70466)
Supplement: Supplementary file 1 — Clean Revised Supplementary Material. [file HEX-28-e70466-s001.docx]

Supplementary materials

[Supplementary file 1a: Initial Program Theory Search in EMBASE and Scopus* 2](#_Toc209009193)

[Supplementary file 1b: Website search 3](#_Toc209009194)

[Supplementary file 2: Guide for interviews with CBT therapists to develop initial program theory 5](#_Toc209009195)

[Supplementary file 3: Therapist, client, and therapy factors found to impact CBT outcomes 6](#_Toc209009196)

[Supplementary file 4: List of all context-mechanism-outcome configurations including sample data 8](#_Toc209009197)

[References 22](#_Toc209009198)

## Supplementary file 1a: Initial Program Theory Search in EMBASE and Scopus*

Embase <1974 to 2023 April 25>

1 ("umbrella review" or "meta-synthesis" or "meta-narrative").ti. 2437

2 review of reviews.ti. 447

3 overview of reviews.ti. 133

4 summar* of review*.ti. 91

5 synthes?s of review*.ti. 148

6 chapter.pt. 57857

7 cochrane.jx. 24682

8 or/1-7 85778

9 exp *cognitive behavioral therapy/ 9578

10 ("cognitive behavio?r*" or "cognitive-behavio?r*").ti. 15522

11 CBT.ti. 2327

12 ("cogniti* therap*" or "cognitive psychotherap*").ti. 2405

13 or/9-12 23207

14 8 and 13 436

15 theoretical model/ or conceptual framework/ 125981

16 (model or models or theor* or concept* or framework* or "middle range" or "mid range" or paradigm* or explanation or origin or definition* or foundation* or principle* or "frame of reference" or framing or construct* or hypothesi*).tw. 8250706

17 15 or 16 8278830

18 (regression model* or model fit or analytical model* or random effects model* or adjusted model* or coefficent model* or co-efficient model* or joint model* or predictive model* or (linear adj2 model*) or (rasch adj3 model*) or (cox adj3 model*)).mp. 658187

19 17 not 18 7646162

20 (effective* or personalis* or personaliz* or tailor* or custom*).ti. 343793

3 and 20 1960

22 17 and 21 501

*This search strategy was translated for use in Scopus.

## Supplementary file 1b: Website search

| **Country** | **Psychology association/ institution** | **Website URL** | **Number of webpages** | |
| --- | --- | --- | --- | --- |
|  |  |  | **Search results for title screening (manual search and search within the website using search terms, e.g. "CBT", "cognitive behaviour therapy", "cognitive behavior therapy")** | **Search results for full-text screening** |
| Canada | International Union of Psychological Science | <https://www.iupsys.net/> | 0 | 0 |
|  | Canadian Agency for Drugs Technologies in Health (CADTH) | <https://www.cadth.ca/> | 316 | 9 |
|  | Canadian Psychological Association (CPA) | <https://cpa.ca/> | 1 | 1 |
|  | Centre for Addiction and Mental Health (CAMH) | <https://www.camh.ca/> | 624 | 2 |
|  | Anxiety Canada | <https://www.anxietycanada.com/> | 0 | 0 |
| U.S | American Psychological Association (APA) | <https://www.apa.org/> | 634 | 34 |
|  | International Association of Applied Psychology | <https://iaapsy.org/> | 0 | 0 |
|  | Social Psychology Network | <https://www.socialpsychology.org/> | 0 | 0 |
|  | International Council of Psychologists | <https://icpweb.org/> | 0 | 0 |
|  | Society of Clinical Psychology (Dvision 12 APA) | <https://div12.org/> | 0 | 0 |
|  | Association for Psychological Science (APS) | <https://www.psychologicalscience.org/> | 54 | 12 |
|  | National Alliance of Professional Psychology Providers | <https://www.nappp.org/> | 0 | 0 |
|  | Association for Behavioral and Cognitive Therapies (ABCT) | <https://www.abct.org/about/core-values-mission-and-vision/> | 100 | 2 |
|  | Beck Institute | https://beckinstitute.org/ | 42 | 23 |
| U.K. | British Psychological Society (BPS) | <https://www.bps.org.uk/> | 1260 | 9 |
|  | British Association for Counselling and Psychotherapy (BACP) | <https://www.bacp.co.uk/> | 699 | 2 |
|  | British Association for Behavioural and Cognitive Psychotherapies (BABCP) | <https://www.babcp.com/> | 518 | 4 |
| Australia | Australian Psychological Society (APS) | <https://psychology.org.au/> | 100 | 1 |
| New Zealand | New Zealand Psychological Society | <https://www.psychology.org.nz/> | 0 | 0 |

## Supplementary file 2: Guide for interviews with CBT therapists to develop initial program theory

*Theory gleaning*

1. What do you find to be the most important outcomes of CBT / for people receiving dialysis suffering from depressive symptoms? [exploring outcomes]
2. What is it about CBT that leads to [above outcomes]? [exploring mechanisms]
3. Does it work like this for everyone? For whom does it work like this and why? For whom does it not work like this? And why do you think that might be? [exploring contexts]
4. How do you assess or evaluate when CBT is “working”? What might be some of the “signposts” you recognize when CBT is “working”? [exploring more outcomes and mechanisms]
5. On the other hand, how do you assess or evaluate when CBT is not “working”? What might be some of the “signposts” you recognize when CBT is not “working?” [exploring more outcomes and mechanisms]
6. Can you please describe for me some of the challenges when CBT does not “work”? [exploring potential unintended outcomes and mechanisms]

*Probing theories*

1. Based on what we found in the literature, the success of CBT depends on [different theories from the literature]. In how far do agree with this? Can you tell us more about your experience/ideas related to this aspect?
2. Another therapist we talked to said [different theories from previous interviews]. In how far do agree with this? Can you tell us more about your experience/ideas related to this aspect?

## Supplementary file 3: Therapist, client, and therapy factors found to impact CBT outcomes

| Factors | Associated with improvements | Associated with no/less improvements | Associations with outcomes not specified |
| --- | --- | --- | --- |
| Intrapersonal factors | Acceptance of the CBT treatment rationale [1]  Marital status: being married [1]  Externalizing coping style [1]  Self-directed coping style [2]  Capacity for flexible, constructive thinking [2]  Capacity to learn [3]  Request for therapy [4]  High learned resourcefulness [5]  Patients who demonstrate increased emotional reactivity that they cannot readily regulate [6]  Comorbidities with depression on Axis II [6]  Presentation of a well-assimilated problem [1] | Severe problems [1]  Cognitive dysfunction [1, 3]  Personality disorder [7]  Comorbid personality disorder [1]  Comorbidities with depression on Axis I [6]  Tendency toward more extreme, stable, and avoidant inferential styles [6]  Dispositional resistance [2]  Marital status: being separated, divorced, widowed, or never married [1]  Pre-treatment perfectionism [5] | Client diversity (ethnicity, sexual orientation, religious beliefs) [1]  Cultural background (values, norms, beliefs) [8]  Different patients need different approaches: e.g., some need a more rationalistic, theory focused approach others an approach that is more focused on a warm and empathetic relationship [9] |
| Interpersonal factors |  | Relationship-oriented reasons for depression [1]  Presence of relationship discord or disturbance [1] |  |
| Environmental factors |  | People who are unemployed and living in socioeconomically deprived circumstances tend to benefit less from psychological treatment as a result of persistent stressors that may be unresolved through therapy (i.e., debt, material deprivation, exposure to crime in the neighbourhood, etc.) but also psychological factors that may not be adequately addressed (i.e., perceived low social status may contribute to demoralization and a sense of a lack of control over one’s life and future) [10]. | Other sociodemographic factors [1] |
| Therapist factors | Adherence to the CBT model [1]  Competence/expertise [1, 5]  Psychologically rather than biologically minded [1]  Clients’ engagement with homework predicts outcome. Moreover, therapists who attend to this aspect of CBT are able to improve clients’ engagement with homework, with associated improvements in outcome [6] |  | Personal characteristics [1] |
| Therapy factors | Concrete and symptom-focussed procedures [5, 6]  Therapy is guided by an individualized understanding of a particular client (through judicious and skilful case formulation) [6]  In patients with long-term conditions: specific focus of the condition [11]  In patients with long-term conditions: use of complementary therapeutic models [11]  In patients with long-term conditions: working collaboratively with health professionals, seeing the patient as a 'whole person' [11]  Language: e.g., with dialysis patients, focus on health promoting benefits of adherence (e.g. Why should I limit foods high in potassium?) rather than on negative consequences of non-adherence (e.g. Why should I not eat foods high in potassium?) [12]  The right timing of therapy sessions (e.g., when patients have energy to engage with therapy yet still feel the need for it) ([13] on patients with severe depression and diurnal variations).  Provision of treatment rationale [1]  Cognitive change early in treatment with CT is necessary for response to CT [5] | Abstract discussions [5, 6]  Aetiology of symptoms biomedical rather than psychological [14]  Therapy fails to take into account the particular challenges of client's illness (this can happen when standard CBT protocols are used in patients with long-term conditions [12]). | Length of the therapy [15] |
| Others | Homework compliance [1, 5] |  | The more severe the disorder, the more experienced the therapist needs to be [16]  Lack of trust, aggressivity, or lack of compliance can be related to interpersonal issues and thus have to be addressed through the therapeutic relationship [5] |

## Supplementary file 4: List of all context-mechanism-outcome configurations including sample data

| CMOCs | Sample data from the included literature and interviews with CBT therapists |
| --- | --- |
| Theory 1: Cognitive changes related to illness and self | |
| CMOC 1: If individuals have multiple chronic health issues (C1) or a terminal illness progression (C2), cognitive restructuring and behavior activation may help them re-conceptualize/re-frame who they are and what they are still capable of doing (M). As a result, they may regain a sense of hope (O1) and control (O2), and feel like a whole person and fully human rather than defined by their illness (O3). | (Interviewee–therapist): The cognitive part is really cognitive reframing and you know, changing their perspective and thinking about their illness really differently …. Instead of comparing yourself constantly to the life that you had anticipated for yourself …, really comparing yourself to what the alternatives might be, can lead you to a perspective of more gratitude, what you have, even though it might not be everything you wanted it to be.  KM: So, it's changing the perception of the illness and what you can do, that is a major part of it, you would say?  (Interviewee–therapist): Yeah, adapting their thinking around that. So maybe you can’t change what they actually do, but changing their perspective around it.  ***  I think when they're feeling hopeless about—I don't think they think of themselves as having a separate psychiatric illness. I think they’re hopeless … because they have a terminal condition. But then, if you can dig into that that there is a depression component to their thinking, and you can do cognitive reframing and get people to engage in life's activities, perhaps not in the same way they might have before but in other meaningful ways, people have a different perspective, like it actually isn't as hopeless, that this isn't a life sentence or a death sentence, that it is a challenging disease to live with, but you can still live with it. And … to me that's really what we're trying to change. (Interviewee–therapist)  ***  … a lot of the times, dialysis takes over their life. They tend to only see themselves as dialysis patients …. they tend to forget that there's other things in their life that they're able to do. So, it's kind of digging in deeper … to get them … having that change of mindset and … helping them see that there is more that they're able to do …. even though they used to maybe … run miles, now they're not able to, but maybe they can still walk, or things like that. Having that change of mindset that just because I can't run anymore doesn't mean that I can't do anything. (Interviewee–therapist)  ***  I think in general … they kind of relearn or realize that it's not only their illness, so, managing more of their life outside of dialysis, and implementing these skills in other parts of their life, maybe using it with family relationships or in work or physical activities, or finding hobbies, relationships. I think they learn to kind of implement these same skills in different areas of their life overall. (Interviewee–therapist)  ***  He had been a professional golfer before and had been an athlete and had lost really much of it, his definition of an ability to be physically active. Now he could still walk and do a number of things which we pushed him on being willing to define as physical activity but you know it's a big ask. I think there are some for whom true cognitive restructuring is beneficial. (Interviewee–therapist) |
| CMOC 2: If individuals have multiple chronic health issues (C1) or a terminal illness progression (C2), identifying aspects of their health and well-being that they can influence may give them a sense of hope (M1) and control (M2), which can reduce depressive symptoms (O). | I think the most important outcome really is that … people who didn't think that they could feel better, begin to feel better, and what felt like an overwhelming disease and treatment, is a bit more manageable. I think that has a lot of really positive effects in people's lives. (Interviewee–therapist)  ***  Well, I think when they're feeling hopeless about— I don't think they think of themselves as having a separate psychiatric illness. I think they’re hopeless … because they have a terminal condition. But then, if you can dig into that that there is a depression component to their thinking, and you can do cognitive reframing and get people to engage in life's activities, perhaps not in the same way they might have before but in other meaningful ways, people have a different perspective, like it actually isn't as hopeless, that this isn't a life sentence or a death sentence, that it is a challenging disease to live with, but you can still live with it. And … to me that's really what we're trying to change. (Interviewee–therapist)  ***  I guess step one is to convince them that what they have is depression, and then step two is that addressing the depression will pay dividends for them. (Interviewee–therapist)  ***  There was a lot of other health issues going on, and a lot of things that he couldn't control. His vision was failing, he had arthritis, he was having difficulty with daily living tasks … a lot of things he couldn't control because of his health. And so, we talked a lot about trying to focus on the things that he could, and changing that. (Interviewee–therapist)  ***  Positive self-judgments about being able to manage and control events and their own emotional reactions are seen as largely responsible for their emotional wellbeing and daily life efficacy. Negative self-judgments are what make them depressed or anxious (Williams 1996). [17] |
| CMOC 3: If individuals have multiple chronic health issues (C), helping them make sense of their illness and its various symptoms (e.g. through psychoeducation) (M) may reduce fears and catastrophic thinking (O1), allow individuals to feel less pathologized (O2), more accepting of themselves and their conditions (O3), and increase an overall sense of illness coherence (O4). | One of the core components of CBT is normalizing … that we are saying basically that this normal, we all have these types of thoughts, we all have these types of these actions and these behaviors that we do that are harmful. So, a big part of it is talking about how normal these things are … really validating and normalizing their experiences. And … that also comes with the accepting … it's normal to have these kind of self-critical thoughts or these type of thoughts or I can understand why I have them. (Interviewee–therapist)  *** The line between illness and depression is unclear to themselves, they don't know often if they are sick or tired, or sick and tired. I guess step one is to convince them that what they have is depression, and then step two is that addressing the depression will pay dividends for them. (Interviewee–therapist)  ***  The way that I think about it is like your illness perception, right understanding what it is that your illness is, and you know what got you here, and what do you need to do to get better, that's really important and you know. (Interviewee–therapist)  ***  Often patients with chronic medical conditions that involve changes in cognition catastrophize or misattribute cognitive impairments to something much more sinister, such as the onset of a dementia process such as Alzheimer disease, which can further negatively impact psychological symptoms and interfere with treatment adherence and overall well-being. Being provided accurate information about the nature, causes, and probable progression of cognitive impairments common in CKD, as well as information about lifestyle factors which may help mitigate cognitive declines such as adherence to medical management, diet, and exercise recommendations may reduce the frequency and severity of catastrophic misinterpretations and psychological symptoms. [18]  ***  CBT … attempts to help clients make sense out of or fabricate a meaning for the ways in which they behave. Formulating such meanings nurtures hope and helps to convince clients that they are not “going crazy,” and that their beliefs are not “pathogenic,” … [19]  ***  When the patient learns the concept of the maladaptive interpretation of emotional states, he or she also learns that emotions are not necessarily dysfunctional per se. Rather, they can read them as a definition of the self or of the world and not as a signal of a problematic scenario. [20] |
| CMOC 4: If the underlying cause of a symptom is physical (e.g., uremic) rather than psychological (C), cognitive restructuring may reach a limit (O) because it does not address the underlying physiological mechanisms (M). | *And I think that for some people … it's hard to know like in reality, what is depression and what is illness. And sometimes time teases that apart and as it turns out, they're just pretty sick, and they're going, you know, they're going through something. And, so their mood is about as optimized as it can be, and the reason why they have high scores or they’re reporting not feeling good is really because of the medical side of what's going on.* (Interviewee–therapist) |
| Theory 2: Experiencing pleasant emotions | |
| CMOC 5: If the lives of people are dominated by managing their illness (C1) and/or if they had to give up previously cherished activities due to their medical conditions (C2), helping them re-engage in activities (e.g. through behavior activation or practical problem solving) can restore joy, vitality, meaning, reward, and mastery (M), thereby reducing depressive symptoms (O1) and improving overall well-being (O2). | I think behavioral activation also doesn't have to be physical, it can be, you know, read for 10 min, it can be, you know, it doesn't have to necessarily take up a lot of energy, just has to be something, maybe, that they enjoy doing that makes them feel good. (Interviewee–therapist)  ***  Many, many dialysis patients just don't have the energy to do much, and all they do is dialysis, and … oddly enough, their dialysis days are their busiest days, because they actually have to get out and go somewhere, and the rest of the time, they're really just not doing much. So, you know, kind of skillfully thinking about what types of activities would they enjoy, and are within their realm of physical abilities, getting them to do that, you know, like the theory goes, you know, without doing good things in your life, you don't feel good. So, I think getting people to just engage in more positive, masterful activities is really powerful. (Interviewee–therapist)  ***  I had this one guy I worked with, … big, burly football player, military fellow, … and he was in a wheelchair, he was an amputee, … he would go out to the porch and watch the hummingbirds at the hummingbird feeder, and … he was transfixed …. and he would talk about it and filling the feeder and the water and the sugar … it was beautiful, and that was a way for him to be engaged in something that was meaningful and valuable to him. (Interviewee–therapist)  ***  I did a lot of CBT work with an individual who had not been able to attend mass, and his faith was an enormous part of his identity and he couldn't physically get there or physically sit through a service. So, CBT was helpful with that to practice and to problem solve and to think about who could give him a ride and practice role-playing. (Interviewee–therapist)  ***  BEHAVIOURAL ACTIVATION This intervention technique is offered to patients with low mood and/or anger to enhance perceptions of control …. Behavioural activation allows individuals to gain a sense of mastery and control over their condition by planning and implementing necessary (e.g. dialysis attendance) and pleasurable tasks (e.g. going to the cinema) (Hopko et al. 2003). Session 5 aims to improve a patient’s sense of control and empowerment over their ESKD and/or psychological distress. It builds on the behavioural activation concepts … [21] |
| CMOC 6: If people experience positive psychological states from behavior activation or behavior change (C), they may continue with these behaviors (O1) because they want to continue feeling good or improve even more (reinforcement) (M). This may create gain spirals (O2). | A lot of the times, … they're like, Okay I tried and it felt good, and so that trying kind of motivates them to continue …. there were participants who started off being able to walk a block, and then they increased it over the weeks, and by the end they were walking several blocks, or they were walking several times a week. And so that was really, life changing for them, because now they're able to be more active and do more things. (Interviewee–therapist)  ***  KM: … so that they make a different kind of experience, and they feel better … and that will motivate them to—  (Interviewee–therapist): Absolutely. Reinforcement of other behavior. So rather than yeah, you know, if we go back to the first school of cognitive behavioural therapy, there’s this is concept of differential reinforcement of other behavior.  ***  People who succeed in making one change, and if it's a psychological change like the mood, they will extrapolate and make other changes in their life more easily. (Interviewee–therapist)  ***  Setting patients up for success with achievable behavioral elements of their treatment will help ensure patients gain a feeling of success and self-efficacy that may further motivate them to remain engaged in psychotherapy and medical management of their condition. [18] |
| CMOC 7: If people experience pain, anxiety, and stress (C), techniques like stretching, progressive muscle relaxation, deep breathing, mindfulness, visualization, and self-affirmation may promote relaxation and other positive psychological states (M) thereby helping the person cope (O) with pain, anxiety, and stress.  Alternatively, these techniques may help individuals cope by distracting them (M2) or giving them a sense of control (M3). | So, we did a lot of relaxation skills … we did imagery exercises and deep breathing. And he really enjoyed those, he found them really helpful, and he said that he could really focus on the imagery exercises, so his thing was kind of going back to his home country and reliving a lot of childhood memories and that really kind of brought back that inner peace … and just kind of made him forget for a little bit the physical pain. (Interviewee–therapist)  ***  KM: What do you think helped them deal with pain?  (Interviewee–therapist): Well, there were a lot of skills in the curriculum that really helped them. Everybody loved– 99% of them loved some version of the mindfulness, whether it was deep breathing, whether it was progressive muscle, relaxation, whether it was the visualization, that was a big hit.  ***  *We would change some– … shift to positive self-talk, which isn't quite the same as changing a belief or a schema but it's a start. And for some that was very helpful. I had a gentleman who … would call them mantras, I changed my mantras …. that was very helpful for him …. he would pull quotes from classic rock and roll, and those lines would be his new mantra, Take it easy, Just gotta take it easy, Don't get so stressed out, which was pretty effective …. I think it was giving him some stress relief and some peace of mind in the moment that then reduced his bodily tension and reduced some of his chronic pain.* (Interviewee–therapist)  ***  Rather than Susan’s parents protecting her by ensuring that she does not experience any triggers that might cause her to feel anxious, I encouraged them to help her build on her ability to cope with situations by teaching her coping skills such as breathing, distracting and focusing on helpful thoughts and actions. [22]  ***  Helping individuals learn relaxation skills can be conveyed as a method of increasing control …. [18] |
| Theory 3: Feeling seen, understood, and accepted | |
| CMOC 8: If therapists show accurate empathy (C), individuals feel seen and understood (M), which strengthens the therapeutic alliance (O).  An experienced therapist may be better able to show accurate empathy. | … if it's not skillfully done, you know you can just miss the mark, and it means nothing to the person as opposed to just a slight tweak. If you get the right thought, the right emotion, you've identified it correctly, then you've really engaged them in something which is vital to them as opposed to I don't know, listening to health information about something that you don't think really applies to you. (Interviewee–therapist)  ***  It's about relationship building, building trust, building that connection, letting them know that I understand them that I hear you, and the more that I can read their mind and show them that you're not alone … you're in good company, … (Interviewee–therapist)  ***  Not that I am reading your mind because I'm not reading your mind, but after this many years, you see the pattern, you kind of know … I'm looking at like, what are those common patterns that I see over and over again …. so just like picking up on the emotional nuances. And then I feel like that gets to the underlying like emotional needs. (Interviewee–therapist) |
| CMOC 9: If therapists show unconditional personal regard (C), individuals feel accepted (M) which strengthens the therapeutic alliance (O1) and lays the ground for in-depth cognitive interventions (O2).  An experienced therapist may be better able to show unconditional positive regard and hold the space for difficult thoughts and emotions. | … if they're nervous to tell me something I'll say, There's really not a lot you could tell me that I haven't heard before, and just being that kind of person that can hear all sorts of really negative thoughts, or self-critical thoughts, and things that people kind of are holding and thinking in their head, … even just the active, saying them out loud, … and then normalizing them, validating them. Not having the reaction of That's crazy, or dismissing it, … I think, is really, really important. And then being able to just kind of sit with that. (Interviewee–therapist)  ***  It allows them to really feel like it's a safe environment, it's somewhere where they can talk about things and they're not going to be judged, they're not going to be criticized …. it helps them to feel safe to feel like they can trust the … therapist … to be able to talk more about these things, get more into it, because only by talking more and getting more into it, can we really understand, get to the core beliefs, the crux of things. (Interviewee–therapist)  ***  I think it provides a safe, like a safe space for the client to practice just to be in there and it shows that they can be open and vulnerable and there is no judgment that, there's just complete unconditional regard. I think it shows the client like what they're capable of, that they can be in the emotions and they're going to be okay, that those emotions are not going to kill them. (Interviewee–therapist)  ***  Sometimes it was too much, sometimes it was too emotional, and so our work was to keep the space safe and appropriate …. everybody on the study was highly skilled, highly trained, and I think that was a real asset of the intervention. There wasn't a green clinician in the group. (Interviewee–therapist) |
| CMOC 10: If the therapist shows that they truly understand the person’s illness context (e.g. by tailoring CBT to the specific challenges and concerns of people undergoing dialysis) (C), individuals feel seen and understood (M). As a result, they gain confidence that their symptoms are not unusual (O1) and that something can be done to make them feel better (O2).  This dynamic may be stronger in people with long-term conditions and medically unexplained symptoms. | (Interviewee–therapist): … and also to kind of incorporate more kidney-disease specific language and examples into the content itself, so that it felt like what they were doing was really something which was designed specifically for people, who are in the same circumstances that they were in …. There are some raw themes here that, I think, need to be made a point of, you don't have to wait for the patient … to bring it up, but you should preemptively bring that up because it makes people feel that you really understand the journey that they've been on.  KM: What does it do to them when you make them feel that you understand their journey?  (Interviewee–therapist): It creates hope that there's an opportunity … that there's a chance that they might feel better because you actually sound like you know what you're talking about.  ***  If you really could spend some time and connecting over that you you've been down this road with other people before, you've seen it before, and you think that it can be helpful, I think that grants some legitimacy and then instills some hope that the treatment might actually be effective. (Interviewee–therapist)  ***  If they believe that they've been understood, and they need some proof that they've been understood from the therapist …. and then he'll say, He's the specialist, so maybe he can help me find a way out of this. (Interviewee–therapist)  ***  There is also evidence that a tailored approach is more effective in people with LTCs (long-term conditions, KM) and MUS (medically unexplained symptoms) or where no clear diagnosis or treatment model exist. [4] |
| CMOC 11: People with kidney failure may not know with whom to talk about the emotional impact of living with kidney failure (C). CBT can provide a safe environment for them to express their fears and worries (M) and as a result, give them a sense of control over these emotions (O1), facilitate their processing and acceptance (O2), or simply experience a sense of relief (O3).  This dynamic may be stronger if individuals lack the capacity to engage in cognitive and behavioral CBT interventions. | I do believe that there is value in the cognitive and behavioral skills. But I also believe very much that there's a lot of value in just the human connection …. Many dialysis patients are extremely isolated and really don't feel like they have anyone that they can say the terrible thoughts that are in their head. They don't want to burden their family members, the doctors don't want to hear it, and nurses and technicians are kind of, It's inappropriate share, they let you know pretty quickly, they don't want to hear about the tough spot that you're in. So, who else can you say “I'm worried that I'm not going to live long enough to see my child graduate or my kids wedding”? They don't have people to share that information with or just unburden themselves with that. So, part of what any therapy for depression with dialysis patients would be to give them an opportunity to speak and to unload their issues as well …. I think at a minimum, it gives them the opportunity, to, you know, have catharsis, to kind of like let it all out, to share it with somebody. (Interviewee–therapist)  ***  We engage in conversation. They begin to talk about their experience. So, rather than running from their experience, they air it. (Interviewee–therapist)  ***  What I tell the patients is that there may be some merit in simply talking about what you're going through here to air your concerns, so the issues don't fester. (Interviewee–therapist)  ***  *We did a lot of active listening. My observation is these individuals, were very lonely, had very little— had lost many of their naturally occurring social networks, whether those would be professional or social or community hobby, even sometimes family, faith.* (Interviewee–therapist)  ***  … maybe they had others to talk about, but they had little opportunity to do so, and it takes some courage to talk about these things. And I think some of them did not want to burden their spouses, burden their kids, their family. We were—, it was literally our job to be burdened. (Interviewee–therapist)  ***  It (psychotherapy, KM) also offers a professional mechanism for social support (“someone to talk to”) and self-disclosure around one’s medical narrative. Emotional expression and the provision of validation and empathy in an interpersonal context is a potent combination for one struggling with renal disease. [18] |
| CMOC 12: If people feel seen, understood, and accepted (C), they reconnect with their humanness (M), which may improve their quality of life (O.  This dynamic may be stronger in people with a terminal illness. | (Interviewee–therapist): What I really wished is that there would be integrated behavioral healthcare, mental health care supports within their dialysis clinics …. I don't know what you would call that, because it's not all about mental illness. But the grief work, the end of life work, the wrestling with hard topics, the levels of anxiety were very high. An integrated behavioral healthcare of some sort within that setting, I think, would have improved quality of life. I don't know that it would have extended life, these individuals are very ill. But their quality of life deserves to be as good as possible for as long as possible.  KM: How do you think that that would have an impact on their quality of life?  (Interviewee–therapist): I think they would feel seen, I think they would feel heard, I think they would feel like a human first and a patient second.  ***  (Interviewee–therapist): I'm not proposing that we integrate true mental health care for psychiatric illness. I'm proposing that we integrate behavioral services for a broadly physically ill group of individuals living with undue stress, strain, fear, anxiety, predominantly due to general medical condition. And so, I think you can find a goodness of fit there where it's provided well enough for everybody as opposed to exquisite specialized care for a pretty small group of people …. It could be done quite well and far more broadly, as if it were integrated into the culture of a clinic.  KM: So rather low scale, simple but integrated.  (Interviewee–therapist): That's my opinion. |
| Theory 4: Capacity to engage with CBT | |
| CMOC 13: Individuals with a low sense of self-efficacy (C) may not believe in their own ability to improve to change and improve their symptoms (M) and therefore not get actively involved with CBT (O).  However, CBT may increase a person’s self-efficacy beliefs. | There's that learned helplessness. But at the same time, I think that can be something that can be worked on in therapy, so that would be part of the goal to help them to work on that sense of like I do have control or there are things I can control or to be able to have that sense of agency. (Interviewee–therapist)  ***  The populations that are, or the qualities that make it challenging is some people are just not psychologically minded. They don't like to think about how they think about things, they just believe that they and their reactions just are built into the fabric of the earth. So, there's nothing that they can change about those things. So that becomes difficult. (Interviewee–therapist)  ***  The difficulties listed by Beck can ultimately be reduced to one: the tension between the patient’s hope for emotional relief without active engagement and the therapist’s task of encouraging the patient to seek relief through active engagement …. The patient underrates his or her capacity to master mental functioning. [20] |
| CMOC 14: Individuals with multimorbidity, who have been receiving much of their treatment in a biomedical setting (C), may no longer believe in their own capacity to improve their health or symptoms without medication and surgery (M) and therefore not get involved with CBT (O). | There is something about people … who are part of hospital systems, and who are part of medical systems that there is more … of a resistance to it, because it is such a medical model, and they've been exposed to a medical model for probably most of their journey … and so I do think there is so much emphasis on … medicine … helping to fix things with medication, and helping to fix things with surgery … Because they've been in that system for so long, it may be harder to switch to How could this be, how could be help like psychologically? (Interviewee–therapist)  ***  This can be tough work, because many of the patients do express their distress somatically and constantly ask for medical help when, in fact, the issues may be more psychological in nature. (Interviewee–therapist)  ***  I mean, there are some individuals who just want the pain fixed. They're not interested in talking … they're interested in a fix. That could be a medication, that could be a cream, that could be a procedure … and they have decided when they think about chronic pain, that is the definition of how to remedy … (Interviewee–therapist) |
| CMOC 15: If individuals with multimorbidity have a long history of unsuccessful treatments (C), they may no longer trust that improvements are possible (M) and thus not get involved with CBT (O). | I found it more difficult to work with those participants who, specifically, not necessarily dialysis, but chronic pain. They had been living with chronic pain, for 10-15 years, they had tried medications, and that didn’t work, and they had tried physical therapy, and that didn’t work, and they had surgeries, and they still had pain. And so, it was really challenging, because I feel like they were already kind of shutting it down before they actually gave it a try. (Interviewee–therapist) |
| CMOC 16: Individuals undergoing dialysis may not engage with CBT (O) because they feel that they do not have enough internal and external resources (M). This may be the case, for example, when individuals struggle to concentrate or engage in cognitive restructuring due to medical issues, medication, and dialysis (C1); have other pressing health concerns due to multimorbidity (C2); lack time for CBT due to multiple doctor appointments (C3); face existential issues due to not being able to work anymore (C4); lack social support (C4), or material resources to apply what they learn in CBT (C5).  The cumulation of multiple difficulties may exacerbate this dynamic. However, certain resources may compensate for the lack of other resources.  If the therapist is able to respond flexibly to the person’s circumstances and needs and adapts session’s delivery and pace, people ~~on~~ receiving dialysis may be able to engage. | It’s difficult at times to bear witness to that when you feel like, with just a little bit of help, or if they had some more support at home, things were structured a little differently, it would just be better. A little more resource, especially for the people that have less resources, sometimes they have just less capacity to kind of structure, their environment to optimize change or optimize health that's hard to sit with. (Interviewee–therapist)  ***  A lot of the times, either they couldn't focus, or they were just so stressed over everything that they were dealing with, or they were too tired, a lot of people were falling asleep during the sessions, or they were too sick. So, during dialysis treatment their blood pressure would drop and they were too sick to continue our session. And so, it was a lot of different challenges. (Interviewee–therapist)  ***  Sometimes they were dealing with so many health issues, so dialysis was just a very small part; they had to manage doctors’ appointments for diabetes and had to see physical therapy and they were hospitalized. And so, there were just a lot of health issues going on where they couldn't really focus on anything else other than just putting out little fires …. it was just difficult for them to focus on anything else other than dealing with all these health issues. (Interviewee–therapist)  ***  And also, sometimes, sometimes not all the time, but sometimes the older participants, were a little more challenging as well. You know, sometimes they weren't able to do a lot because they were in in nursing homes. So, they had very limited things that they felt they could do. (Interviewee–therapist)  ***  *I had this participant who I was working with, who had been on dialysis for 15 years. No social support, so, there's no family, a few friends, but not really, you know somebody that he can kind of lean on and he just, you know, he just kept going for 15 years. There was a time where he was homeless. He lost his job. So, he, you know, he didn't have any support, he was sleeping on the streets, and he would just continue to go to treatment and try to keep working and so he said, I think the fact that I'm by myself like that I have nobody to look after me makes me keep going, because you know, I'm all I have, and so I want to continue this for myself. I feel like, you know, a lot of people don't have that. So, it was just more of a personality thing for him like he had this, you know, I just have to keep going, you know that mindset I feel like makes a difference.* (Interviewee–therapist)  ***  You cannot do cognitive behavioral therapy with somebody who's severely depressed …. They don't have the cognitive focus or people who are intoxicated, they usually cancel the therapy session, they don't have the cognitive focus …. and feeling safe …. if they feel unsafe, they may not have the cognitive focus. So, when it's all so complex—. (Interviewee–therapist)  ***  Kidney patients often experience fluctuating health status; as symptoms flare, treatment plans change, or comorbid medical conditions arise. Problems such as fatigue, pain, or nausea may interfere in a patient’s ability to keep scheduled appointments. Last-minute medical procedures or physician visits may get scheduled, or as mentioned above, dialysis treatments may limit a patient’s availability. Consequently, patients may not always be in a position to provide the standard 24- or 48-h notice to cancel appointments. Furthermore, kidney disease patients may have more frequently shifting priorities in therapy based on new or unexpected symptoms, procedures, or treatment plans. [18] |
| CMOC 17: If CBT appointments and delivery are handled flexibly (C1) and goals and tasks are adapted and modified session by session to accommodate the individual’s capacities, and current needs (C2), CBT will likely be more effective (O). The reason is that individuals have the capacity to focus, collaborate, and actively engage with the activities (M). Delivering CBT chairside may accommodate people’s lack of time due to high treatment burden and hence enhance engagement with CBT. | You understand that dialysis itself is a really challenging treatment. And so, we would stop the session, and either give them the option to try and add another session, another day or sometimes we would call at home if they were up for it. Just, you know, kind of giving them a lot of options to try to reschedule and continue the session at a later time, when they were feeling better. (Interviewee–therapist)  ***  Also giving them the option of having therapy, while they're on dialysis, because, like, I mentioned a lot of dialysis patients have so many other medical appointments that therapy is just one more appointment, and they may not be willing to do it outside of dialysis. I feel like it's easier to have that option of like, I'm sitting here 3hs, I can spare an hour for therapy, right? You get more success, giving them that option of, Hey, I can be chairside right? Or I could be, you know on a tablet talking to you, one-on-one while you're here, right while you're receiving your treatment. (Interviewee–therapist) |
| Theory 5: Normalizing conversations about mental health | |
| CMOC 18: If individuals hold or perceive stigma around mental health issues (C), they may be reluctant to get involved with CBT (O), because they worry that they could be perceived as weak or weird (M).  Stigma may be more prevalent in certain ethnic communities (e.g., Hispanic and Asian) and within these communities among older generations (C). | There's people that really just don't believe in it, don't think it's helpful. There's a stigma, and there's a resistance to coming to therapy, to having to work on those issues, and sometimes that can be a challenge. (Interviewee–therapist)  ***  I worked a lot with the Latino population, and specifically the older patients were more doubtful, maybe, of having the need for therapy …. I heard a lot of, I'm not crazy, I don't need therapy … it's more of a stigma. (Interviewee–therapist)  ***  … there's a tremendous stigma associated. I've had people come and talk with me from the Asian culture who said that it's a sign of weakness, you simply don't talk about psychological struggles. Yeah. So, certainly we need to be aware of the socio-cultural context in which people are coming from. (Interviewee–therapist) |
| CMOC 19: In an integrated kidney care setting (C), individuals might be reluctant to disclose their emotional struggles (e.g., by signing up for CBT) (O) because they worry that they could be perceived as complicated or ungrateful which may negatively affect their overall care (M).  OR  In an integrated kidney care setting (C) individuals may sign up for CBT and let others know (O) because they think that taking proactive steps to manage their health creates a positive impression leading to better overall care (M).  People’s decisions may be influenced by whether they, or their kidney care environment, perceive mental health issues as stigma. | So, I think for some people, they don't want the whole care team involved in their mental health business …. I think that a lot of people have a pretty specific relationship with their care team. They want to be seen as a good patient, and they want to get good care, and the way to do that is to not be difficult in any way. And somebody who is sad or dissatisfied with what life is offering them, in their mind, is perceived as being difficult or ungrateful, and therefore they don't want their care team to know that. They specifically don't want their nurses and doctors to know that they're struggling emotionally, even if it has nothing to do with dialysis. (Interviewee–therapist)  ***  Patients may be less willing to divulge information to a psychotherapist knowing that a full team of care providers may be privy to that information. [18]  ***  He was not sure if he wanted his nephrologist and everyone else to know he would be seeing a psychologist, but after discussing the pros and cons, he decided it might make a good impression on his other healthcare providers if they saw that he was taking steps to manage things better. [18] |
| CMOC 20: If conversations about mental health are incorporated into routine dialysis care (C1) and/or individuals are referred to a CBT therapist by their trusted medical experts (C2), they may consider CBT when needed (O). This is because they consider it as acceptable to talk about their mental health (M1) and/or trust the expert’s opinion (M2). | Starting just when you meet with your team at the beginning, you meet with a nurse and a doctor, but then also having maybe not a psychologist, but a social worker or someone within that mental health realm, right from the beginning, just to normalize, Hey, we recognize that this also has an impact on your mental health and how you feel and just checking in. (Interviewee–therapist)  ***  They felt very strongly that their doctors didn't want to discuss it, that they were given the message that this wasn't the place for it. So, they really wanted to kind of normalize the discussion of depression. (Interviewee–therapist)  ***  What is really exciting for me is that the front-end work is now being done by the endocrinologists and by other members of the team, because it's safe for people to talk with the dietician about dietary work, it's safe for them to talk with the endocrinologist about their … hemoglobin and their medication. And if these people whom they respect and trust, then say, Well, look, there's something else here you might want to consider …. Sometimes it's hard to talk about some of the struggles that you have, but it could be helpful. Would you consider it? And some people will say, Well, not right now, but in many cases, they do come in due course. (Interviewee–therapist)  ***  Kidney disease treatment programs that can provide access to mental health services should consider normalizing this process early on in the CKD journey by incorporating discussions about mental health and provide consultation with mental health specialists into routine multidisciplinary care. [18] |
| CMOC 21: If individuals hold stigma related to mental health issues (C), avoiding labels such as “psychotherapy” and “depression” may increase their acceptance of CBT (O) as they no longer feel pathologized (M). | People didn't like some of the pathologizing words that CBT sometimes used. People don't like ‘depression,’ they don't like that word, they don't wanna think of it as a psychiatric illness or a mental health condition. They didn't like those terms, they wanted to use less stigmatizing language. (Interviewee–therapist)  ***  You could call it ‘stress,’ you could call it ‘support,’ you could call it ‘coaching,’ you could call it ‘life coaching.’ (Interviewee–therapist) |

## References

1. Dimidjian S, Dobson KS. Processes of change in cognitive therapy. In: Reinecke MA, Clark MA (eds). Cognitive Therapy across the Lifespan. Cambridge University Press; 2003:477-506.

2. Clark DA, Reinecke MA. Cognitive therapy in the twenty-first century: Current status and future directions. In: Reinecke MA, Clark DA (eds). Cognitive Therapy across the Lifespan: Evidence and Practice2003:507-28.

3. Hollon SD, De Rubeis RJ, Andrews PW, et al. Cognitive therapy in the treatment and prevention of depression: A fifty-year retrospective with an evolutionary coda. Cognitive Therapy and Research. 2020;**45**(3):402-17. doi:10.1007/s10608-020-10132-1.

4. Schulte D. Tailor-made and standardized therapy: complementary tasks in behavior therapy. A contrarian view. J Behav Ther Exp Psychiatry 1996;**27**(2):119-26. doi:10.1016/0005-7916(96)00015-8.

5. Blackburn I-M, Moorhead S. Update in cognitive therapy for depression. Journal of Cognitive Psychotherapy. 2000;**14**(3):305-36. doi:10.1891/0889-8391.14.3.305.

6. Kuyken W, Dalgleish T, Holden ER. Advances in cognitive-behavioural therapy for unipolar depression. Can J Psychiatry. 2007;**52**(1):5-13. doi:10.1177/070674370705200103.

7. Williams RM. Cognitive therapy for difficult patients - a review. International Review of Psychiatry. 1994;**6**:175-86.

8. Rathod SK, D. Cognitive behaviour therapy across cultures. Psychiatry. 2009;**8**(9):370-1.

9. Boucher T. Cognitive‐behavioural contributions to pluralistic practice: Reflections on an issue of some contention. Therapy and Beyond2010:155-69.

10. Delgadillo J, Rubel J, Barkham M. Towards personalized allocation of patients to therapists. J Consult Clin Psychol. 2020;**88**(9):799-808. doi:10.1037/ccp0000507.

11. Sanders S, Coppin S, Moulson H, et al. What adaptions are effective to cognitive behavioural interventions for adults with long-term conditions and medically unexplained symptoms? A systematic review. Ansiedad y Estrés. 2020;**26**(2-3):188-201. doi:10.1016/j.anyes.2020.07.002.

12. Hudson JL, Moss-Morris R, Game D, et al. Improving distress in dialysis (iDiD): A tailored CBT self-management treatment for patients undergoing dialysis. Journal of Renal Care. 2016;**42**(4):223-38. doi:10.1111/jorc.12168.

13. Frais AT. Could positive diurnal variations in severe depression be the key factor for delivering effective cognitive behaviour therapy? Med Hypotheses. 2009;**72**(6):677-8. doi:10.1016/j.mehy.2009.01.027.

14. Geraghty KJ, Blease C. Cognitive behavioural therapy in the treatment of chronic fatigue syndrome: A narrative review on efficacy and informed consent. J Health Psychol. 2018;**23**(1):127-38. doi:10.1177/1359105316667798.

15. Dobson K, Dobson D. Empirically supported treatments: Recent developments in the cognitive-behavioural therapies, and implications for evidence-based psychotherapy. In: Loewenthal D, Winter D (eds). What is Psychotherapeutic Research? 1st ed. Routledge; 2006.

16. Beck AT. The current state of cognitive therapy: A 40-year retrospective. Archives of General Psychiatry. 2005;**62**(9):953-9. doi:10.1001/archpsyc.62.9.953.

17. Ruggiero GM, Spada MM, Caselli G, et al. A historical and theoretical review of cognitive behavioral therapies: From structural self-knowledge to functional processes. J Ration Emot Cogn Behav Ther. 2018;**36**(4):378-403. doi:10.1007/s10942-018-0292-8.

18. Tulloch TG, King JP, Pellizzari JR, et al. Overview of psychotherapy principles for patients with kidney disease. In: Hategan A, Bourgeois JA, Gangji AS, W. WTK (eds). Psychonephrology: A Guide to Principles and Practice. Springer Nature Switzerland AG; 2022:105-29.

19. Meichenbaum D. Evolution of cognitive behavior therapy: Origins, tenets, and clinical examples. In: Zeig JK (ed). The Evolution of Psychotherapy: The Second Conference. Routledge; 1992:114-22.

20. Ruggiero GM, Caselli G, Sassaroli S. Case formulation in standard cognitive therapy. In: Ruggiero GM, Caselli G, Sassaroli S (eds). CBT Case Formulation as Therapeutic Process Springer Nature Switzerland AG; 2021:17-33.

21. Hudson JL, Moss‐Morris R, Game D, et al. Improving Distress in Dialysis (iDiD): A tailored CBT self-management treatment for patients undergoing dialysis. Journal of Renal Care. 2016;**42**(4):223-38. doi:10.1111/jorc.12168.

22. Hayes C. Clinical psychology: A psychoeducational cognitive behavioural approach to helping people cope. In: Devonport TJ (ed). Managing Stress: From Theory to Application. Nova Science Publishers; 2012:229-58.
